# A Population-Based Study of Bariatric Surgery Trends in Australia: Variations Reflect Continuing Inequities in Access to Surgery

**DOI:** 10.1007/s11695-025-07699-7

**Published:** 2025-02-06

**Authors:** Thomas Goubar, Christopher Goubar, Douglas Fenton-Lee, Aneta Stefanidis, Peter S. Macdonald, R. Louise Rushworth

**Affiliations:** 1 St Vincent’s Hospital Sydney, Darlinghurst, Australia; 2 The University of Notre Dame Australia, Fremantle, Australia; 3 Deakin University, Geelong, Australia

**Keywords:** Bariatric surgery, Healthcare disparities, Obesity treatment, Public vs private healthcare, Socioeconomic barriers

## Abstract

**Background:**

Obesity is increasingly prevalent and associated with higher morbidity and mortality. Bariatric surgery, particularly sleeve gastrectomy, provides durable weight loss and improves obesity-related conditions like type 2 diabetes and cardiovascular disease. Despite its benefits, significant concerns regarding inequities in access to bariatric surgery persist. This study aims to evaluate recent trends in bariatric surgery rates and to investigate patterns of access to bariatric surgery.

**Methods:**

A population-based study of age-adjusted bariatric procedure rates in adults in NSW, Australia, was conducted over the financial years 2013/14 to 2021/22. Trends in age-adjusted procedure rates were assessed by demographics and healthcare settings.

**Results:**

In 2021/22, 179.6 bariatric procedures per 100,000 population were performed, an 89.7% increase since 2013/14 (*p* < 0.001). Laparoscopic sleeve gastrectomy, the most common procedure, increased by 94.1% to 112.6/100,000/year (*p* < 0.001). Females had 3.6 times higher rates than males (232.3/100,000/year vs 64.9/100,000/year). The greatest increases occurred in younger patients (125.9% in the 18–24 age group; 142.4% in the 25–34 age group, *p* < 0.001). Private hospital rates were 15.6 times higher than public (132.2/100,000/year vs 8.5/100,000/year) and rose 92.3% (*p* < 0.001), whilst public hospital rates declined by 17.9% (p = NS). Patients from regional areas had the highest rates (175.7/100,000/year) and largest increase (169.8%; 89.4/100,000/year to 241.8/100,000/year, *p* < 0.001).

**Conclusions:**

Bariatric surgery rates continue to increase, particularly among females, despite similar obesity prevalence between sexes. Most surgery is conducted in the private sector, suggesting differential access based on financial circumstances. Rates are highest in younger people, although obesity-related comorbidities increase with age. Regional patients undergo surgery at higher rates than rural patients despite greater obesity prevalence with increasing rurality. Efforts to address these disparities are essential to improve equitable access to obesity treatments.

**Supplementary Information:**

The online version contains supplementary material available at 10.1007/s11695-025-07699-7.

## Introduction

Obesity affects approximately 20% of adults worldwide, and its prevalence continues to rise [1]. Obesity contributes significantly to the global burden of chronic disease, with cardiovascular disease being the leading cause of obesity-related death [1, 2]. While obesity rates are generally higher in women globally, rates in Australia are comparable between men and women (33% vs. 31%) [2, 3]. The growing prevalence of obesity has increased the demand for effective therapeutic strategies, which range from lifestyle modifications and pharmacotherapy to surgical interventions.

Bariatric surgery is an effective and durable treatment for obesity that significantly reduces obesity-related cardiovascular morbidity and mortality [4, 5]. Initially indicated for weight loss in individuals with class II obesity, bariatric surgery is now used to manage comorbid metabolic conditions and has innovative applications in high surgical-risk patients, such as those with heart failure and obesity, where surgery acts as a bridge to heart transplant [6–8]. Concurrently, pharmacological therapies, including glucagon-like peptide receptor agonists (GLP-1RA), have emerged as viable treatments for obesity and obesity-related cardiovascular disease, reshaping the therapeutic landscape [9, 10].

Since its inception, bariatric surgery has become an increasingly common procedure in Australia, with laparoscopic sleeve gastrectomy (LSG) being preferred [11–13]. Its widespread adoption reflects its favourable safety profile, shorter procedure time, and lower complication rate compared to Roux-en-Y gastric bypass (RYGB), which has comparable weight loss outcomes [14–16].

Despite its benefits and cost-effectiveness, bariatric surgery remains inaccessible to many individuals, with significant inequities linked to socioeconomic status, geographical location, and availability of public healthcare services. These inequities are influenced by financial barriers, limited availability of services in rural areas, and stricter eligibility criteria in public hospitals, further exacerbating disparities in care [17, 18]. However, there is limited information on bariatric surgery trends over recent years, according to patient demographics and healthcare settings, to determine if these inequities persist. This study aims to address this shortfall by investigating recent trends in bariatric surgery rates in New South Wales (NSW), Australia, over nine years, focusing on demographics and healthcare settings.

## Methods

### Study Design and Setting

The design of this study was a population-based analysis of trends in bariatric procedure rates and obesity prevalence over nine financial years (2013/14 to 2021/22). Data were obtained from the Admitted Patient Data Collection (APDC), a census of all admitted patient data provided to the NSW Ministry of Health by NSW public and private hospitals reported by Australian financial year. The APDC contains data on up to 50 diagnoses and 50 procedures relevant to each episode of hospitalisation. Procedures are coded using the Australian Classification of Health Interventions (ACHI).

### Study Population

The study cohort included all adults aged 18 years or older admitted to NSW hospitals by financial year (July 1 to June 30) between 2013/14 and 2021/22, including a code for bariatric surgery (n = 81,492). Patients with missing data (< 0.1% of records, n = 73) were excluded. For each financial year, the study included approximately 6 million adults, representing the NSW hospital-treated population [19]. This study adheres to The Strengthening the Reporting of Observational Studies in Epidemiology (STROBE) guidelines [20].

### Demographic Variables

De-identified patient data included age, sex, hospital type (public or private), local health district of residence, year of admission, and any procedures performed during that admission. Age was categorised into six groups: 18–24, 25–34, 35–44, 45–54, 55–64, and 65 + years. Procedure codes were grouped according to surgical intention (primary and revision) (Supporting Information: Table S1). Geographical comparisons were assessed using the NSW local health district of residence, categorised as metropolitan, regional and rural (Supporting information: Table S2).

### Outcomes

The primary outcome was the direct age-adjusted rate of bariatric procedures per 100,000 population. Trends in the rates were assessed by age, sex, hospital type and geography.

### Statistical Analysis

Direct age-adjusted procedure rates were calculated using the NSW population in 2013 as the base. All rates reported are age-adjusted rates, except those in age-specific categories. Rates were calculated using NSW population estimates and specific local health district population estimates [19, 21]. NSW obesity estimates were obtained using NSW Population Health Survey data published by the Centre for Epidemiology and Evidence [22]. Rates and confidence intervals were calculated using R Statistical Software (Version 4.3.1). Given the size of the population sample, rate differences were assessed using a conservative confidence interval [CI] of 99.9%, presented in square brackets throughout the manuscript and a corresponding *p*-value (*p* < 0.001). Ethics approval was provided by an institutional Human Research Ethics Committee (2020-128S).

## Results

### Total Procedures

**Fig. 1:**
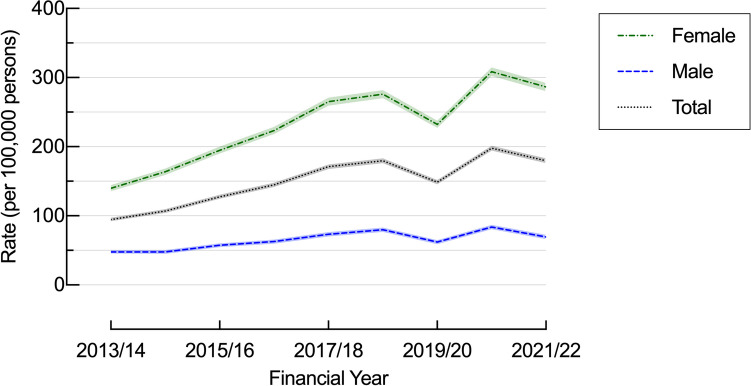
Age-adjusted bariatric surgery rates per 100,000 population by sex, NSW, 2013/14 to 2021/2022

Between 2013/14 and 2021/22, 81,419 bariatric procedures were performed in NSW, averaging 150.1 procedures/100,000/year. These increased by 89.7%, from 94.7/100,000/year [99.9% CI: 90.4 – 99.0] to 179.6/100,000/year [174.0 – 185.2] over the study (Table 1 & Fig. 1). Of the total procedures, 67,817 (83.3%) were primary bariatric surgeries, and 13,602 (16.7%) were revision procedures. Primary procedures were 5.0 times more common than revision procedures (125.0/100,000/year [123.4 – 126.6] vs 25.1/100,000/year [24.4 – 25.8]) and increased to a greater extent, 99.3% (76.4/100,000/year [72.6 – 80.2] to 152.3/100,000/year [147.1 – 157.5]) compared to a 49.2% increase in revision procedures (18.3/100,000/year [16.5 – 20.1] to 27.3/100,000/year [25.1 – 29.5]) (Table 2). Endoscopic procedures were rare (7.7/100,000/year [7.3 – 8.1]) but increased by 2140.0% (0.5/100,000/year [0.2 – 0.8] to 11.2/100,000/year [9.8 – 12.6]).

**Table 1:** Demographic characteristics of adult patients undergoing obesity procedures, NSW, 2013/14 to 2021/22 (N = 81,419)

	Procedures		NSW population#		Procedure estimates*	Obesity estimates*
Demographic	*n*	%	*n*	%	Rate/100,000/year (99.9% CI)	Rate/100/year (99.9% CI)
Total	81,419		5,733,451		150.1 (148.3, 151.8)	29.8 (29.8, 29.8)
Age group (years)						
18–24	4,438	5.5	699,326	12.2	68.5 (65.1, 71.9)	15.9 (15.9, 16.0)
25–34	17,181	21.1	1,061,341	18.5	164.7 (160.6, 168.9)	23.3 (23.2, 23.4)
35–44	23,608	29	1,019,028	17.8	248.2 (242.9, 253.7)	29.9 (29.8, 29.9)
45–54	21,507	26.4	981,041	17.1	238.9 (233.5, 244.3)	34.7 (34.7, 34.8)
55–64	11,522	14.1	852,623	14.9	139.8 (135.5, 144.1)	37.5 (37.4, 37.5)
65 +	3,163	3.9	1,120,092	19.5	27.9 (26.3, 29.6)	34.4 (34.3, 34.4)
Sex						
Female	64,155	78.8	2,917,764	50.9	232.3 (229.3, 235.3)	29.2 (29.1, 29.2)
Male	17,264	21.2	2,815,687	49.1	64.9 (63.3, 66.5)	30.6 (30.5, 30.6)
Setting						
Public hospital	4,620	6.1			8.5 (8.1, 8.9)	
Private hospital	71,685	93.9			132.2 (130.5, 133.8)	
Geography						
Metropolitan	46,278	62.4	3,377,075	60.9	142.7 (140.6, 144.9)	19.1 (17.6, 20.7)
Regional	19,523	26.3	1,201,388	21.7	175.7 (171.6, 179.9)	25.7 (23.4, 28.0)
Rural	8,348	11.3	964,024	17.9	94.8 (91.4, 98.2)	31.2 (26.7, 35.7)

#Population estimates for 2013

*Average rates for study period

**Table 2:** Age-adjusted and age-specific rates for bariatric surgery by age-group and sex, NSW, 2013/14 to 2021/22 (N = 81,419)

		Average	2013/14		2021/22			
Characteristic		Rate/100,000/year	Rate/100,000	95% CI	Rate/100,000	95% CI	% Change	*P Value*
Procedures		150.1	94.7	90.4—99.0	179.6	174.0—185.2	89.7	< 0.001
18–24		68.5	41.3	33.2—49.5	93.4	81.1—105.6	125.9	< 0.001
25–34		164.7	94.2	84.4—104.0	228.4	213.7—243.1	142.4	< 0.001
35–44		248.2	154.5	141.5—167.4	291.5	274.5—308.4	88.7	< 0.001
45–54		238.9	143.0	130.3—155.7	277.4	260.2—294.7	94.0	< 0.001
55–64		139.8	106.5	94.7—118.2	143.6	130.8—156.4	34.8	< 0.001
65 +		27.9	22.9	18.2—27.7	27.2	22.5—31.9	18.5	NS
Females		232.3	140.0	132.7—147.3	286.4	276.4—296.4	104.6	< 0.001
18–24		118.5	67.6	52.6—82.5	163.5	140.1—186.8	141.9	< 0.001
25–34		269.3	148.7	131.1—166.4	381.9	355.1—408.8	156.8	< 0.001
35–44		388.7	235.6	213.1—258.0	461.9	431.8—492.0	96.1	< 0.001
45–54		366.8	211.8	190.0—233.5	438.8	408.3—469.4	107.2	< 0.001
55–64		208.9	149.7	130.0—169.3	225.7	203.2—248.2	50.8	< 0.001
65 +		36.4	25.7	18.8—32.7	39.8	32.1—47.5	54.6	NS
Males		64.9	47.8	43.5—52.1	69.4	64.4—74.4	45.2	< 0.001
18–24		21.2	16.2	8.9—23.5	28.3	18.8—37.8	74.5	NS
25–34		60.0	39.7	30.6—48.9	75.1	63.2—87.1	89.1	< 0.001
35–44		106.1	71.6	59.0—84.2	119.1	103.7—134.6	66.3	< 0.001
45–54		107.0	72.6	59.7—85.5	112.2	96.4—128.0	54.5	< 0.001
55–64		67.7	62.2	49.4—75.1	58.0	46.3—69.8	−6.7	NS
65 +		18.2	19.7	13.2—26.3	12.9	8.2—17.6	−34.6	NS
Primary		125.0	76.4	72.6—80.2	152.3	147.1—157.5	99.3	< 0.001
Females		191.5	110.3	103.8—116.8	241.3	232.1—250.5	118.8	< 0.001
Males		56.1	41.4	37.4—45.4	60.5	55.8—65.2	46.3	< 0.001
Revision		25.1	18.3	16.5—20.1	27.3	25.1—29.5	49.2	< 0.001
Females		40.8	29.7	26.3—33.1	45.0	41.1—48.9	51.5	< 0.001
Males		8.7	6.4	4.8—8.0	8.9	7.1—10.7	38.7	NS
Endoscopic		7.7	0.5	0.2—0.8	11.2	9.8—12.6	2140.0	< 0.001
Females		12.2	0.6	0.1—1.1	17.7	15.2—20.2	2850.0	< 0.001
Males		3.1	0.4	0.0—0.8	4.4	3.1—5.7	1000.0	< 0.001
Laparoscopic Sleeve Gastrectomy		98.7	58.0	54.7—61.3	112.6	108.2—117.0	94.1	< 0.001
Females		150.6	82.9	77.4—88.4	178.5	170.6—186.4	115.3	< 0.001
Males		44.2	32.2	28.7—35.7	44.6	40.6—48.6	38.5	< 0.001
Laparoscopic gastric bypass		15.2	2.7	1.9—3.5	29.5	27.2—31.8	992.6	< 0.001
Females		23.6	3.8	2.6—5.0	46.7	42.7—50.7	1128.9	< 0.001
Males		6.5	1.4	0.6—2.2	11.8	9.7—13.9	742.9	< 0.001
Laparoscopic removal of gastric band		12.1	9.0	7.7—10.4	9.5	8.2—10.8	5.6	NS
Females		19.5	14.7	12.3—17.1	15.4	13.1—17.7	4.8	NS
Males		4.4	3.1	2.0—4.2	3.4	2.2—4.6	9.7	NS

LSG comprised 65.7% (n = 53,521) of all procedures (average 98.7/100,000/year [97.3 – 100.1]) and increased by 94.1% (58.0/100,000/year [54.7 – 61.3] to 112.6/100,000/year [108.2 – 117.0]) (Table 2). In contrast, laparoscopic gastric bypass was 6.5 times less common (average 15.2/100,000/year [14.6 – 15.7]) than LSG but increased to a greater extent (992.6%, 2.7/100,000/year [1.9 – 3.5] to 29.5/100,000/year [27.2 – 31.8]). The most common revision procedure was laparoscopic removal of gastric banding, representing 48.3% of revision procedures (12.1/100,000/year [11.6 – 12.6]), increasing by 72.7% from 9.0/100,000/year [7.7 – 10.4] in 2013/14 to 15.6/100,000/year [14.0 – 17.3] in 2017/18, and subsequently declining to 9.5/100,000/year [8.2 – 10.8] by 2021/22 (Table 2).

### Sex Differences

The majority (78.5%, n = 59,910) of patients having bariatric surgery were female, with a procedure rate of 232.3/100,000/year [229.3 – 235.3] compared to 64.9/100,000/year [63.3 – 66.5] in males (Fig. 1) (Table 1). Rates increased in females by 104.6% (140.0/100,000/year [132.7 −147.3] to 286.4/100,000/year [276.4 – 296.4]) and to a lesser extent in males, by 45.2%, from 47.8/100,000/year [43.5 – 52.1] to 69.4/100,000/year [64.4 – 74.4] (Fig. 1). LSG rates were 3.4 times higher in females (150.6/100,000/year [148.2 – 153.1] vs 44.2/100,000/year [43.5 – 46.2]) and increased by 115.3% in females (82.9/100,000/year [77.4 – 88.4] to 178.5/100,000/year [170.6 – 186.4]), and by 38.5%, (32.3/100,000/year [28.7 – 35.7] to 44.6/100,000/year [40.6 – 48.6]) in males (Table 2).

### Age Differences

**Fig. 2:**
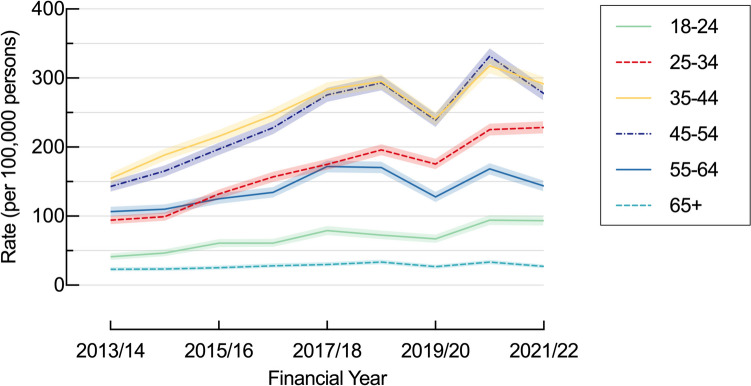
Age-specific bariatric surgery rates per 100,000/year population by age group, NSW, 2013/14 to 2021/22

Procedure rates increased with age, to be highest in middle age and decreased thereafter (18–24: 68.5/100,000/year [65.1 – 71.9]; 35–44: 248.2/100,000/year [242.9 – 253.6]; 65 + : 27.9/100,000/year [26.3 – 29.6]) (Table 2). However, the most significant increases were observed in the youngest age groups, with the magnitude of the rise diminishing with advancing age (18–24: 125.9%; 35–44: 88.7%; 65 + : 18.5%) (Fig. 2) (Table 2). LSG rates were highest in those aged 35–44 years (168.9/100,000/year [164.5 – 173.3]). However, the greatest increase was identified in the 25–34 year age group (165.3%, from 60.6/100,000/year [52.6 – 68.6] to 160.7/100,000/year [148.4 – 173.0]) while there was a progressively smaller increase with each increase in age category and rates were stable rates in those aged 55–64 years (23.4%, 61.0/100,000/year [52.1 – 69.9] to 75.2/100,000/year [65.9 – 84.6]) and 65 + years (9.6/100,000/year [6.4 – 12.7] to 10.2/100,000/year [7.3 – 13.0]) (Supporting Information: Table S3).

### Age-Sex Specific Differences

Procedure rates increased across younger age groups in females, being greatest in those aged 25–34 years (156.8%, 148.7/100,000/year [131.1 – 166.4] to 381.9/100,000/year [355.1 – 408.8]) and stable in those aged 65 + years (54.6%, 25.7/100,000/year [18.8 – 32.7] to 39.8/100,000/year [32.1 – 47.5]) (Table 2). In contrast, male rates increased in middle-aged groups, with stable rates in those 18–24 years (74.5%, 16.2/100,000/year [8.9 – 23.5] to 28.3/100,000/year [18.8 – 37.8]), 55–64 years (−6.7%, 62.2/100,000/year [49.4 – 75.1] to 58.0/100,000/year [46.3 – 69.8]) and 65 + years (−34.6%, 19.7/100,000/year [13.2 – 26.3] to 12.9/100,000/year [8.2 – 17.6]) (Table 2). Rates for LSG increased significantly in females across all age groups, particularly the youngest age groups (18–24: 184.8%, 43.3/100,000/year [31.3 – 55.3] to 123.3/100,000/year [103.0 – 143.6]), but did not change in those aged 65 + years (36.3%, 10.8/100,000/year [6.2 – 15.4] to 14.7/100,000/year [10.0 – 19.4]). By comparison, in males, LSG rates did not change in the youngest age group (18–24: 75.4%, 11.2/100,000/year [5.1 – 17.2] to 19.6/100,000/year [11.7 – 27.6]), nor in the oldest age-groups (65 + : −37.6%, 8.1/100,000/year [3.7 – 12.5] to 5.1/100,000/year [2.0 – 8.1]) while they increased among the middle-aged groups (Supporting Information: Table S3).

### Healthcare Setting Differences

**Fig. 3:**
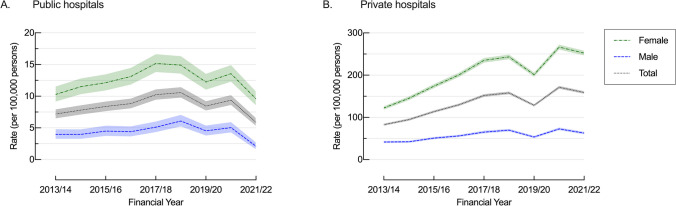
Age-adjusted bariatric surgery rates per 100,000 by sex in (**A**) public and (**B**) private hospitals, NSW, 2013/14 to 2021/22

Admission rates to private hospitals were more than 15.5 times higher than those in public hospitals (132.1/100,000/year [130.5 – 133.8] vs 8.5/100,000/year [8.1 – 8.9]) (Table 1). Public hospital rates remained stable (−17.9%, 7.2/100,000/year [6.0 – 8.4] to 5.9/100,000/year [4.9 – 6.9]), whilst private hospital rates increased by 92.3% (82.7/100,000/year [78.8 – 86.6] to 159.0/100,000/year [153.7 – 164.3]) (Fig. 3). Public hospital rates were stable in both males (47.5%, 4.0/100,000/year [2.7 – 5.3] to 2.1/100,000/year [1.2 – 3.0]) and females (10.3/100,000/year [8.4 – 12.2] and 9.6/100,000/year [7.8 – 11.4]). Meanwhile, private hospital rates increased by 106.4% in females, from 122.2/100,000/year [115.5 – 128.9] to 252.2/100,000/year [242.8 – 261.6]) with a smaller increase among males (50.0%, 41.8/100,000/year [37.8 – 45.8] to 62.7/100,000/year [57.9 – 67.5]). In regards to age differences, private hospital rates increased to a greater extent in younger age groups (18–24: 142.1%; 35–44: 89.8%; 65 + : 10.6%) and, conversely, public hospital rates declined to a greater extent in the youngest age-groups (18–24: −53.4%; 35–44: −39.4%; 65 + : 60.7%) these changes were not significant (Supporting Information: Table S3).

### Geographical Differences

**Fig. 4:**
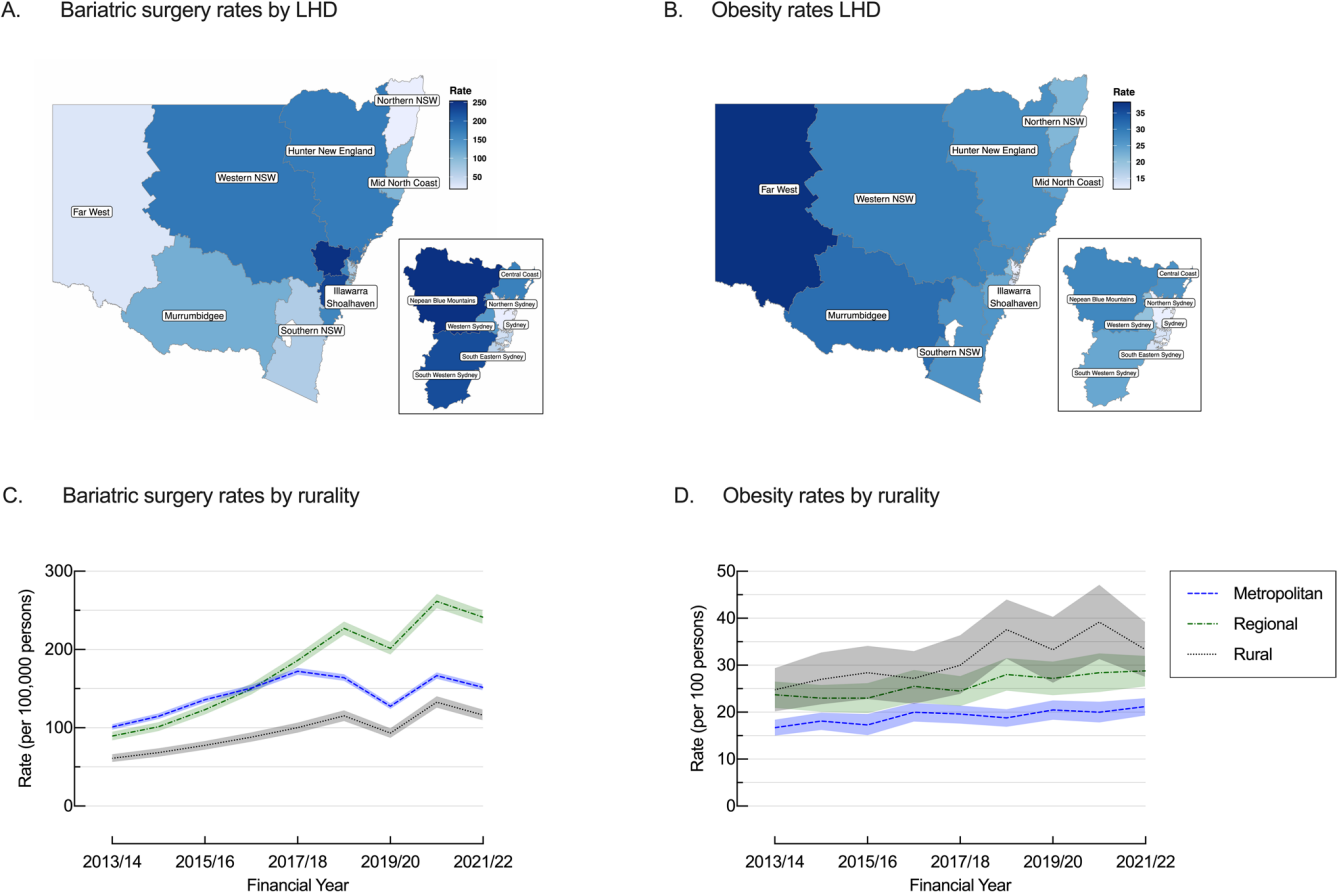
Age-adjusted (**A**) average bariatric surgery rates per 100,000 population by local health district (LHD), (**B**) average obesity rates per 100 population by LHD; (**C**) bariatric surgery rates per 100,000 population by geographical area and (**D**) obesity rates per 100 population by geographical area, NSW, 2013/14 to 2021/22

Bariatric surgery was performed more commonly among patients from regional areas (175.7/100,000/year [173.2 – 178.2]), followed by metropolitan areas (142.7/100,000/year [141.4 – 144.0]), while the rate among patients from rural areas was nearly half that for regional areas (94.8/100,000/year [92.7 – 96.8]) (Table 1) (Fig. 4). Despite this, average obesity prevalence was highest in rural (31.2% [26.7 – 35.7]), followed by regional (25.7% [23.4 – 28.0]) areas and lowest in metropolitan (19.1% [17.6 – 20.7]) areas (Table 1) (Fig. 4).

Over the study period, bariatric surgery increased for patients from all areas. Regional areas experienced the greatest increase by 169.8%; from 89.4/100,000/year [80.3 – 98.5] to 241.2/100,000/year [226.7 – 255.7]), followed by rural areas (89.9%; 61.2/100,000/year [52.9 – 69.5] to 116.2/100,000/year [104.9 – 127.5]), and metropolitan areas (50.1%; 101.0/100,000/year [95.3 −106.7] to 151.6/100,000/year [145.0 – 158.2]) (Table 3) (Fig. 4). At the same time, obesity rates increased in all areas, with rural areas experiencing the greatest increase (34.3% increase; 24.8% [20.2 – 29.4] to 33.3% [27.5 – 39.1]), followed by metropolitan (26.9% increase; 16.7% [15.0 – 18.4] to 21.2% [19.3 – 23.0]) and regional (21.5% increase; 23.7% [20.9 – 26.5] to 28.8% [25.5 – 32.0]) (Fig. 4).

**Table 3:** Age-adjusted bariatric surgery rates by healthcare setting and geography, NSW, 2013/14 to 2021/22 (N = 81,419)

		Average	2013/14		2021/22			
Characteristic		Rate/100,000/year	Rate/100,000	99.9% CI	Rate/100,000	99.9% CI	% Change	*P Value*
Public hospital		8.5	7.2	6.0—8.4	5.9	4.9—6.9	−17.9	NS
Females		12.5	10.3	8.4—12.2	9.6	7.8—11.4	−6.8	NS
Males		4.4	4.0	2.7—5.3	2.1	1.2—3.0	−47.5	NS
Private hospital		132.2	82.7	78.8—86.6	159.0	153.7—164.3	92.3	< 0.001
Females		204.5	122.2	115.5—128.9	252.2	242.8—261.6	106.4	< 0.001
Males		57.2	41.8	37.8—45.8	62.7	57.9—67.5	50.0	< 0.001
Metropolitan		142.7	101.0	95.3—106.7	151.6	145.0—158.2	50.1	< 0.001
Females		216.1	146.9	137.2—156.6	235.3	223.6—247.0	60.2	< 0.001
Males		66.9	53.5	47.5—59.5	65.6	59.3—71.9	22.6	NS
Regional		175.7	89.4	80.3—98.5	241.2	226.7—255.7	169.8	< 0.001
Females		277.0	136.2	120.5—151.9	388.9	363.2—414.6	185.5	< 0.001
Males		68.9	40.3	31.6—49.0	86.4	73.9—98.9	114.4	< 0.001
Rural		94.8	61.2	52.9—69.5	116.2	104.9—127.5	89.9	< 0.001
Females		149.9	91.3	76.9—105.7	193.7	173.1—214.3	112.2	< 0.001
Males		37.2	30.0	21.5—38.5	35.8	26.6—45.0	19.3	NS

## Discussion

This large population-based study of bariatric surgery reveals significant changes and disparities in bariatric surgery rates over recent years. Overall, bariatric procedures were performed more often, with LSG being the most common, although laparoscopic gastric bypass rates also increased. Bariatric surgery is performed more frequently in women, and the disparity between the sexes increased despite a comparable obesity prevalence [3]. Similarly, surgery was generally more common in younger people. It increased disproportionately in these age groups despite obesity prevalence increasing with age, together with age-related increases in obesity-related comorbidities [22]. Geographical disparities also persist, with rural patients, whose obesity prevalence is highest, having the lowest procedure rates [17]. Most procedures were undertaken in the private sector, suggesting that previously identified financial factors continue to influence health-related decision-making in obesity management [17, 18].

New pharmacological treatments for obesity, such as GLP-1RAs, have altered the landscape of obesity management [9]. Currently, three GLP-1RA products are approved for weight loss in Australia by the Therapeutic Goods Administration (TGA): liraglutide (approved 2018), semaglutide (approved 2022), and tirzepatide (approved September 2024). However, significant supply shortages since then have restricted their availability, potentially limiting their impact on bariatric surgery trends during the study period. While these treatments are highly effective, their long-term effect on the demand for bariatric surgery remains unclear [23]. Pharmacotherapy may either reduce the need for surgery or complement surgical approaches, particularly in populations for whom surgery is contraindicated or delayed [24]. This phenomenon has been observed recently in the United States, where increased GLP-1RA usage coincided with a decline in bariatric surgery between 2022 and 2023 [25]. However, not all patients can tolerate these drugs, and they are often expensive and difficult to access [10, 26].

The gender gap in bariatric surgery rates is well-established, and our findings show that this gap has widened over time [27]. Despite similar obesity prevalence between men and women in Australia, women are significantly more likely to undergo bariatric surgery. This trend is not unique to Australia, but the extent of the difference suggests that additional factors beyond clinical need may be influencing access to surgery [28]. Significant and complex gendered social factors, including differing perceptions of excess weight and surgery, health-seeking behaviours, and referral rates, impact sex-specific bariatric surgery rates [29]. While bariatric surgery is equally effective for both sexes, the lower rate of surgery among men suggests a better understanding of the barriers to accessing treatment is required [30].

Procedure rates have grown disproportionately in younger age groups, particularly among those aged 25–34 years, while there have been more modest increases among older individuals despite the higher prevalence of obesity and related comorbidities in older populations [31]. This underutilisation of bariatric surgery in older adults represents a missed opportunity, as growing evidence supports the safety and efficacy of surgery in this group, showing significant improvements in weight reduction and comorbidity management, often with complication rates comparable to younger patients [32–34]. The lower surgery rates in older age groups may reflect outdated perceptions of surgical risk, limited awareness of the benefits, or systemic barriers such as under-referral and access issues [35]. Given the aging population and the substantial burden of obesity-related conditions in older adults, bariatric surgery should be considered a viable treatment option for weight and obesity-related comorbidity management in older people.

In NSW, most bariatric procedures are performed in private hospitals, with a noticeable decline in public hospital procedures over the study period. While public hospital surgical rates increased between 2013/14 and 2018/19, this trend reversed in 2019/20 with the onset of the COVID-19 pandemic, likely reflecting deferrals and delays in elective procedures. The predominance of surgery provision in private hospitals reflects the influence of financial resources on access to bariatric surgery rather than differences in outcomes [18]. National data demonstrate that individuals from lower socioeconomic backgrounds are more likely to experience obesity but face more significant challenges in accessing surgical interventions due to the predominance of private sector services [17, 36].

This analysis identified that a higher proportion of older individuals and men undergo bariatric surgery in public hospitals, likely reflecting a greater burden of cardiovascular comorbidities and high-risk cases, such as those using surgery as a bridge to transplant [37]. This disparity suggests that the current healthcare system disproportionately benefits those with greater financial means, leaving underserved populations without adequate access to beneficial treatments [38]. Financial factors also limit access to pharmacological therapies among many who may benefit, further exacerbating socioeconomic disparities. Addressing these inequities will require public healthcare policy reforms to improve access to weight loss interventions for lower-income groups.

Geographical disparities in bariatric surgery rates are considerable and persist, with regional areas exhibiting the highest rates, followed by metropolitan areas, with rural areas, despite having the highest obesity prevalence, experiencing the lowest surgery rates [17]. This suggests that there are significant barriers to access for rural populations, who arguably stand to benefit the most from bariatric surgery. The relative absence of specialised centres, greater travel distances, and limited healthcare infrastructure in rural areas likely contribute to these differences [39, 40]. The greater rise in surgery rates in regional areas compared to metropolitan areas suggests some improvement in access, but rural populations remain underserved.

### Limitations

This study comprises a large population-based analysis of bariatric surgery trends in Australia; however, limitations exist. First, the analysis was restricted to data from 2013/14 onwards, as before this, bariatric surgery was coded using codes outside ACHI Block 889 (obesity procedures) and, therefore, could not be compared to modern ACHI codes. Second, the COVID-19 pandemic may have impacted surgery rates in later years due to deferrals of elective procedures. Third, the analysis of geographical trends was based on the local health district of residence only, and whilst highlighting populations of need, it could not assess geographical areas of bariatric surgery provision. Fourth, the dataset did not include information on patients' financial circumstances, such as insurance status or out-of-pocket costs, which may also contribute to inequities in access to bariatric surgery. Despite these limitations, this study establishes significant disparities in bariatric surgery trends.

### Conclusion

In conclusion, bariatric surgery, especially LSG, has become more common, with younger individuals and women being more likely to undergo surgery despite high obesity rates and comorbidities among older populations and men. The predominance of bariatric surgery in private hospitals highlights the influence of financial barriers: lower socioeconomic and rural areas appear to be underserved. Geographic disparities, particularly the underutilisation of surgery in patients from rural regions, underscore the need for targeted healthcare policy reforms. As new pharmacological treatments such as GLP-1RA continue to reshape obesity management, future research should explore their long-term effects on bariatric surgery trends and address barriers to ensure equitable access for all populations.

## Supplementary Information

Below is the link to the electronic supplementary material.

Supplementary file1 (DOCX 68 KB)

## Data Availability

The de-identified data we analysed are not publicly available, but requests to the corresponding author for the data will be considered case-by-case.

## Declarations

### Data Sharing Statement

The de-identified data we analysed are not publicly available, but requests to the corresponding author for the data will be considered case-by-case.
