# Supplementary material for: A Population-Based Study of Bariatric Surgery Trends in Australia: Variations Reflect Continuing Inequities in Access to Surgery
Source: Obes Surg. 2025 Feb 6;35(3):1026–35. doi: 10.1007/s11695-025-07699-7 (PMC11906563; doi:10.1007/s11695-025-07699-7)
Supplement: Supplementary file 1 — Supplementary file1 (DOCX 68 KB) [file 11695_2025_7699_MOESM1_ESM.docx]

**Appendix/Supplement**

| **Table S1** **Age-adjusted and age-sex specific rates for bariatric surgery by age-group and sex, NSW, 2013/14 to 2021/22 (N= 81,419)** | | | | | | | | |
| --- | --- | --- | --- | --- | --- | --- | --- | --- |
|  |  | **Average** | **2013/14** | | **2021/22** | |  |  |
| **Characteristic** | | **Rate/100,000/year** | **Rate/100,000** | **99.9% CI** | **Rate/100,000** | **99.9% CI** | **% Change** | ***P Value*** |
| Procedures | | 150.1 | 94.7 | 90.4 - 99.0 | 179.6 | 174.0 - 185.2 | 89.7 | < 0.001 |
|  | 18-24 | 68.5 | 41.3 | 33.2 - 49.5 | 93.4 | 81.1 - 105.6 | 125.9 | < 0.001 |
|  | 25-34 | 164.7 | 94.2 | 84.4 - 104.0 | 228.4 | 213.7 - 243.1 | 142.4 | < 0.001 |
|  | 35-44 | 248.2 | 154.5 | 141.5 - 167.4 | 291.5 | 274.5 - 308.4 | 88.7 | < 0.001 |
|  | 45-54 | 238.9 | 143.0 | 130.3 - 155.7 | 277.4 | 260.2 - 294.7 | 94.0 | < 0.001 |
|  | 55-64 | 139.8 | 106.5 | 94.7 - 118.2 | 143.6 | 130.8 - 156.4 | 34.8 | < 0.001 |
|  | 65+ | 27.9 | 22.9 | 18.2 - 27.7 | 27.2 | 22.5 - 31.9 | 18.5 | NS |
| Females | | 232.3 | 140.0 | 132.7 - 147.3 | 286.4 | 276.4 - 296.4 | 104.6 | < 0.001 |
|  | 18-24 | 118.5 | 67.6 | 52.6 - 82.5 | 163.5 | 140.1 - 186.8 | 141.9 | < 0.001 |
|  | 25-34 | 269.3 | 148.7 | 131.1 - 166.4 | 381.9 | 355.1 - 408.8 | 156.8 | < 0.001 |
|  | 35-44 | 388.7 | 235.6 | 213.1 - 258.0 | 461.9 | 431.8 - 492.0 | 96.1 | < 0.001 |
|  | 45-54 | 366.8 | 211.8 | 190.0 - 233.5 | 438.8 | 408.3 - 469.4 | 107.2 | < 0.001 |
|  | 55-64 | 208.9 | 149.7 | 130.0 - 169.3 | 225.7 | 203.2 - 248.2 | 50.8 | < 0.001 |
|  | 65+ | 36.4 | 25.7 | 18.8 - 32.7 | 39.8 | 32.1 - 47.5 | 54.6 | NS |
| Males | | 64.9 | 47.8 | 43.5 - 52.1 | 69.4 | 64.4 - 74.4 | 45.2 | < 0.001 |
|  | 18-24 | 21.2 | 16.2 | 8.9 - 23.5 | 28.3 | 18.8 - 37.8 | 74.5 | NS |
|  | 25-34 | 60.0 | 39.7 | 30.6 - 48.9 | 75.1 | 63.2 - 87.1 | 89.1 | < 0.001 |
|  | 35-44 | 106.1 | 71.6 | 59.0 - 84.2 | 119.1 | 103.7 - 134.6 | 66.3 | < 0.001 |
|  | 45-54 | 107.0 | 72.6 | 59.7 - 85.5 | 112.2 | 96.4 - 128.0 | 54.5 | < 0.001 |
|  | 55-64 | 67.7 | 62.2 | 49.4 - 75.1 | 58.0 | 46.3 - 69.8 | -6.7 | NS |
|  | 65+ | 18.2 | 19.7 | 13.2 - 26.3 | 12.9 | 8.2 - 17.6 | -34.6 | NS |
| Primary | | 125.0 | 76.4 | 72.6 - 80.2 | 152.3 | 147.1 - 157.5 | 99.3 | < 0.001 |
|  | 18-24 | 62.9 | 35.5 | 27.9 - 43.1 | 86.7 | 74.9 - 98.5 | 144.4 | < 0.001 |
|  | 25-34 | 146.3 | 79.7 | 70.6 - 88.8 | 205.7 | 191.8 - 219.6 | 158.0 | < 0.001 |
|  | 35-44 | 211.3 | 128.0 | 116.2 - 139.8 | 251.6 | 235.8 - 267.4 | 96.6 | < 0.001 |
|  | 45-54 | 194.2 | 113.3 | 102.1 - 124.5 | 231.5 | 215.7 - 247.3 | 104.2 | < 0.001 |
|  | 55-64 | 106.8 | 80.7 | 70.5 - 90.9 | 109.6 | 98.4 - 120.8 | 35.9 | < 0.001 |
|  | 65+ | 18.3 | 16.5 | 12.4 - 20.6 | 15.7 | 12.2 - 19.2 | -4.9 | NS |
| Females | | 191.5 | 110.3 | 103.8 - 116.8 | 241.3 | 232.1 - 250.5 | 118.8 | < 0.001 |
|  | 18-24 | 108.4 | 57.6 | 43.8 - 71.4 | 152.3 | 129.7 - 174.9 | 164.3 | < 0.001 |
|  | 25-34 | 237.6 | 122.2 | 106.2 - 138.2 | 343.1 | 317.7 - 368.5 | 180.9 | < 0.001 |
|  | 35-44 | 326.7 | 190.1 | 170.0 - 210.2 | 395.2 | 367.2 - 423.2 | 107.9 | < 0.001 |
|  | 45-54 | 294.5 | 163.4 | 144.3 - 182.5 | 363.8 | 335.9 - 391.7 | 122.6 | < 0.001 |
|  | 55-64 | 156.2 | 108.7 | 92.0 - 125.4 | 167.9 | 148.4 - 187.4 | 54.6 | < 0.001 |
|  | 65+ | 22.8 | 18.9 | 12.9 - 24.9 | 22.5 | 16.6 - 28.4 | 19.0 | NS |
| Males | | 56.1 | 41.4 | 37.4 - 45.4 | 60.5 | 55.8 - 65.2 | 46.3 | < 0.001 |
|  | 18-24 | 20.0 | 14.3 | 7.4 - 21.2 | 25.8 | 16.7 - 34.9 | 80.8 | NS |
|  | 25-34 | 54.8 | 37.3 | 28.4 - 46.2 | 68.5 | 57.1 - 79.9 | 83.6 | < 0.001 |
|  | 35-44 | 94.6 | 64.5 | 52.6 - 76.4 | 106.4 | 91.7 - 121.1 | 65.0 | < 0.001 |
|  | 45-54 | 90.8 | 62.1 | 50.2 - 74.0 | 96.0 | 81.4 - 110.6 | 54.6 | < 0.001 |
|  | 55-64 | 55.2 | 52.0 | 40.2 - 63.8 | 48.9 | 38.1 - 59.7 | -6.1 | NS |
|  | 65+ | 13.3 | 13.7 | 8.2 - 19.2 | 8.0 | 4.2 - 11.8 | -41.9 | NS |
| Revision | | 25.1 | 18.3 | 16.5 - 20.1 | 27.3 | 25.1 - 29.5 | 49.2 | < 0.001 |
|  | 18-24 | 5.5 | 5.9 | 2.7 - 9.1 | 6.7 | 3.3 - 10.1 | 14.1 | NS |
|  | 25-34 | 18.4 | 14.5 | 10.6 - 18.4 | 22.7 | 18.0 - 27.4 | 56.8 | NS |
|  | 35-44 | 36.9 | 26.5 | 21.0 - 32.0 | 39.9 | 33.5 - 46.3 | 50.5 | < 0.001 |
|  | 45-54 | 44.7 | 29.7 | 23.9 - 35.5 | 46.0 | 38.9 - 53.1 | 55.0 | < 0.001 |
|  | 55-64 | 33.0 | 25.8 | 20.0 - 31.6 | 34.0 | 27.7 - 40.3 | 31.6 | NS |
|  | 65+ | 9.6 | 6.4 | 3.8 - 9.0 | 11.5 | 8.5 - 14.5 | 78.6 | NS |
| Females | | 40.8 | 29.7 | 26.3 - 33.1 | 45.0 | 41.1 - 48.9 | 51.5 | < 0.001 |
|  | 18-24 | 10.1 | 9.9 | 4.0 - 15.8 | 11.2 | 4.9 - 17.5 | 12.4 | NS |
|  | 25-34 | 31.7 | 26.6 | 19.1 - 34.1 | 38.8 | 30.2 - 47.4 | 46.1 | NS |
|  | 35-44 | 62.0 | 45.4 | 35.4 - 55.4 | 66.8 | 55.2 - 78.4 | 46.8 | NS |
|  | 45-54 | 72.3 | 48.4 | 37.9 - 58.9 | 75.1 | 62.3 - 87.9 | 55.2 | < 0.001 |
|  | 55-64 | 52.7 | 41.0 | 30.7 - 51.3 | 57.8 | 46.3 - 69.3 | 40.8 | NS |
|  | 65+ | 13.6 | 6.8 | 3.2 - 10.4 | 17.3 | 12.2 - 22.4 | 153.7 | < 0.001 |
| Males | | 8.7 | 6.4 | 4.8 - 8.0 | 8.9 | 7.1 - 10.7 | 38.7 | NS |
|  | 18-24 | 1.2 | 2.0 | 0.0 - 4.7 | 2.5 | 0.0 - 5.5 | 28.9 | NS |
|  | 25-34 | 5.1 | 2.4 | 0.0 - 4.8 | 6.7 | 3.1 - 10.3 | 173.3 | NS |
|  | 35-44 | 11.5 | 7.1 | 3.0 - 11.2 | 12.7 | 7.5 - 17.9 | 78.2 | NS |
|  | 45-54 | 16.2 | 10.5 | 5.5 - 15.5 | 16.2 | 10.1 - 22.3 | 53.7 | NS |
|  | 55-64 | 12.5 | 10.2 | 4.8 - 15.6 | 9.2 | 4.3 - 14.1 | -10.2 | NS |
|  | 65+ | 4.9 | 6.0 | 2.3 - 9.7 | 4.9 | 2.0 - 7.8 | -18.1 | NS |
| Endoscopic | | 7.7 | 0.5 | 0.2 - 0.8 | 11.2 | 9.8 - 12.6 | 2140.0 | < 0.001 |
|  | 18-24 | 4.6 | 0.4 | 0.0 - 1.4 | 7.0 | 3.6 - 10.4 | 1650.0 | < 0.001 |
|  | 25-34 | 10.5 | 0.5 | 0.0 - 1.3 | 19.1 | 14.8 - 23.4 | 3720.0 | < 0.001 |
|  | 35-44 | 13.6 | 0.6 | 0.0 - 1.5 | 18.9 | 14.5 - 23.3 | 3050.0 | < 0.001 |
|  | 45-54 | 11.2 | 0.7 | 0.0 - 1.7 | 13.8 | 9.9 - 17.7 | 1871.4 | < 0.001 |
|  | 55-64 | 4.9 | 0.8 | 0.0 - 2.0 | 5.9 | 3.2 - 8.6 | 637.5 | < 0.001 |
|  | 65+ | 0.7 | 0.0 | 0.0 - 0.3 | 0.9 | 0.0 - 1.8 | NA | NS |
| Females | | 12.2 | 0.6 | 0.1 - 1.1 | 17.7 | 15.2 - 20.2 | 2850.0 | < 0.001 |
|  | 18-24 | 8.6 | 0.6 | 0.0 - 2.3 | 12.7 | 5.9 - 19.5 | 2016.7 | < 0.001 |
|  | 25-34 | 17.6 | 0.9 | 0.0 - 2.5 | 31.1 | 23.3 - 38.9 | 3355.6 | < 0.001 |
|  | 35-44 | 21.4 | 0.8 | 0.0 - 2.3 | 28.8 | 21.2 - 36.4 | 3500.0 | < 0.001 |
|  | 45-54 | 17.2 | 0.6 | 0.0 - 2.0 | 21.4 | 14.5 - 28.3 | 3466.7 | < 0.001 |
|  | 55-64 | 7.5 | 0.9 | 0.0 - 2.7 | 10.4 | 5.4 - 15.4 | 1055.6 | < 0.001 |
|  | 65+ | 1.0 | 0.0 | 0.0 - 0.5 | 1.2 | 0.0 - 2.6 | NA | NS |
| Males | | 3.1 | 0.4 | 0.0 - 0.8 | 4.4 | 3.1 - 5.7 | 1000.0 | < 0.001 |
|  | 18-24 | 0.8 | 0.3 | 0.0 - 1.6 | 1.7 | 0.0 - 4.3 | 466.7 | NS |
|  | 25-34 | 3.4 | 0.0 | 0.0 - 0.6 | 7.2 | 3.4 - 11.0 | NA | < 0.001 |
|  | 35-44 | 5.9 | 0.4 | 0.0 - 1.6 | 8.9 | 4.5 - 13.3 | 2125.0 | < 0.001 |
|  | 45-54 | 5.0 | 0.8 | 0.0 - 2.4 | 6.0 | 2.2 - 9.8 | 650.0 | NS |
|  | 55-64 | 2.2 | 0.7 | 0.0 - 2.3 | 1.1 | 0.0 - 2.9 | 57.1 | NS |
|  | 65+ | 0.4 | 0.0 | 0.0 - 0.6 | 0.5 | 0.0 - 1.5 | NA | NS |
| Laparoscopic Sleeve Gastrectomy | | 98.7 | 58.0 | 54.7 - 61.3 | 112.6 | 108.2 - 117.0 | 94.1 | < 0.001 |
|  | 18-24 | 52.5 | 26.9 | 20.3 - 33.4 | 69.5 | 58.9 - 80.1 | 158.6 | < 0.001 |
|  | 25-34 | 119.9 | 60.6 | 52.6 - 68.6 | 160.7 | 148.4 - 173.0 | 165.3 | < 0.001 |
|  | 35-44 | 168.9 | 99.7 | 89.4 - 110.0 | 185.4 | 171.9 - 198.9 | 86.0 | < 0.001 |
|  | 45-54 | 149.4 | 86.8 | 76.9 - 96.8 | 165.1 | 151.8 - 178.5 | 90.1 | < 0.001 |
|  | 55-64 | 80.8 | 61.0 | 52.1 - 69.9 | 75.2 | 65.9 - 84.6 | 23.4 | NS |
|  | 65+ | 12.7 | 9.6 | 6.4 - 12.7 | 10.2 | 7.3 - 13.0 | 6.7 | NS |
| Females | | 150.6 | 82.9 | 77.4 - 88.4 | 178.5 | 170.6 - 186.4 | 115.3 | < 0.001 |
|  | 18-24 | 90.9 | 43.3 | 31.3 - 55.3 | 123.3 | 103.0 - 143.6 | 184.8 | < 0.001 |
|  | 25-34 | 194.3 | 92.8 | 78.8 - 106.7 | 269.4 | 246.8 - 292.0 | 190.4 | < 0.001 |
|  | 35-44 | 259.7 | 145.4 | 127.8 - 163.1 | 292.7 | 268.7 - 316.7 | 101.2 | < 0.001 |
|  | 45-54 | 224.8 | 123.7 | 107.1 - 140.3 | 255.8 | 232.3 - 279.2 | 106.8 | < 0.001 |
|  | 55-64 | 117.3 | 81.5 | 67.1 - 96.0 | 114.3 | 98.2 - 130.4 | 40.1 | < 0.001 |
|  | 65+ | 15.6 | 10.8 | 6.2 - 15.4 | 14.7 | 10.0 - 19.4 | 36.3 | NS |
| Males | | 44.2 | 32.2 | 28.7 - 35.7 | 44.6 | 40.6 - 48.6 | 38.5 | < 0.001 |
|  | 18-24 | 16.3 | 11.2 | 5.1 - 17.2 | 19.6 | 11.7 - 27.6 | 75.4 | NS |
|  | 25-34 | 45.4 | 28.4 | 20.6 - 36.2 | 52.2 | 42.2 - 62.1 | 83.4 | < 0.001 |
|  | 35-44 | 77.0 | 53.0 | 42.1 - 63.8 | 76.9 | 64.5 - 89.3 | 45.2 | < 0.001 |
|  | 45-54 | 71.7 | 49.1 | 38.4 - 59.8 | 72.3 | 59.6 - 84.9 | 47.2 | NS |
|  | 55-64 | 42.7 | 39.9 | 29.6 - 50.2 | 34.6 | 25.5 - 43.6 | -13.4 | NS |
|  | 65+ | 9.4 | 8.1 | 3.7 - 12.5 | 5.1 | 2.0 - 8.1 | -37.6 | NS |
| Laparoscopic gastric bypass | | 15.2 | 2.7 | 1.9 - 3.5 | 29.5 | 27.2 - 31.8 | 992.6 | < 0.001 |
|  | 18-24 | 4.1 | 0.7 | 0.0 - 2.0 | 10.6 | 6.4 - 14.8 | 1384.9 | < 0.001 |
|  | 25-34 | 13.2 | 2.8 | 1.1 - 4.6 | 29.7 | 24.4 - 35.0 | 950.8 | < 0.001 |
|  | 35-44 | 24.4 | 4.5 | 2.2 - 6.8 | 50.0 | 42.9 - 57.0 | 1007.6 | < 0.001 |
|  | 45-54 | 28.7 | 3.9 | 1.7 - 6.1 | 52.1 | 44.5 - 59.6 | 1244.5 | < 0.001 |
|  | 55-64 | 16.8 | 3.4 | 1.2 - 5.6 | 27.4 | 21.8 - 33.0 | 705.0 | < 0.001 |
|  | 65+ | 2.6 | 0.4 | 0.0 - 1.0 | 4.4 | 2.4 - 6.3 | 1125.6 | < 0.001 |
| Females | | 23.6 | 3.8 | 2.6 - 5.0 | 46.7 | 42.7 - 50.7 | 1128.9 | < 0.001 |
|  | 18-24 | 6.4 | 1.5 | 0.0 - 3.9 | 17.2 | 9.4 - 25.0 | 1077.6 | < 0.001 |
|  | 25-34 | 21.7 | 5.1 | 1.7 - 8.4 | 48.5 | 38.9 - 58.0 | 851.9 | < 0.001 |
|  | 35-44 | 38.3 | 6.4 | 2.5 - 10.3 | 77.7 | 65.2 - 90.2 | 1112.1 | < 0.001 |
|  | 45-54 | 45.0 | 4.6 | 1.2 - 8.1 | 85.4 | 71.8 - 99.0 | 1742.6 | < 0.001 |
|  | 55-64 | 25.4 | 4.9 | 1.2 - 8.6 | 42.4 | 32.5 - 52.3 | 771.4 | < 0.001 |
|  | 65+ | 3.4 | 0.5 | 0.0 - 1.7 | 6.5 | 3.3 - 9.7 | 1200.0 | < 0.001 |
| Males | | 6.5 | 1.4 | 0.6 - 2.2 | 11.8 | 9.7 - 13.9 | 742.9 | < 0.001 |
|  | 18-24 | 1.9 | 0.0 | 0.0 - 0.8 | 4.5 | 0.5 - 8.4 | NA | NS |
|  | 25-34 | 4.7 | 0.6 | 0.0 - 1.9 | 11.0 | 6.4 - 15.6 | 1843.2 | < 0.001 |
|  | 35-44 | 10.3 | 2.6 | 0.1 - 5.1 | 22.0 | 15.3 - 28.7 | 753.2 | < 0.001 |
|  | 45-54 | 12.0 | 3.1 | 0.2 - 5.9 | 18.0 | 11.5 - 24.4 | 480.6 | < 0.001 |
|  | 55-64 | 7.7 | 1.9 | 0.0 - 4.3 | 11.7 | 6.3 - 17.2 | 517.7 | < 0.001 |
|  | 65+ | 1.6 | 0.2 | 0.0 - 1.1 | 2.0 | 0.1 - 3.9 | 931.8 | NS |
| Laparoscopic removal of gastric band | | 12.1 | 9.0 | 7.7 - 10.3 | 9.5 | 8.2 - 10.8 | 5.6 | NS |
|  | 18-24 | 1.5 | 2.4 | 0.3 - 4.5 | 1.3 | 0.0 - 2.9 | -46.2 | NS |
|  | 25-34 | 7.2 | 7.6 | 4.8 - 10.5 | 4.5 | 2.4 - 6.7 | -40.4 | NS |
|  | 35-44 | 16.2 | 12.5 | 8.8 - 16.2 | 10.9 | 7.6 - 14.3 | -12.2 | NS |
|  | 45-54 | 21.8 | 15.2 | 11.0 - 19.4 | 16.0 | 11.8 - 20.2 | 5.2 | NS |
|  | 55-64 | 18.7 | 13.4 | 9.1 - 17.7 | 15.9 | 11.6 - 20.2 | 18.8 | NS |
|  | 65+ | 6.2 | 2.6 | 0.9 - 4.3 | 7.4 | 4.9 - 9.9 | 185.5 | < 0.001 |
| Females | | 19.5 | 14.7 | 12.3 - 17.1 | 15.4 | 13.1 - 17.7 | 4.8 | NS |
|  | 18-24 | 2.6 | 4.4 | 0.4 - 8.3 | 2.1 | 0.0 - 5.1 | -51.8 | NS |
|  | 25-34 | 12.2 | 14.1 | 8.6 - 19.7 | 7.0 | 3.3 - 10.8 | -50.2 | NS |
|  | 35-44 | 27.5 | 21.4 | 14.6 - 28.2 | 18.9 | 12.6 - 25.2 | -11.6 | NS |
|  | 45-54 | 35.2 | 26.0 | 18.3 - 33.7 | 26.1 | 18.6 - 33.7 | 0.5 | NS |
|  | 55-64 | 29.6 | 20.9 | 13.5 - 28.2 | 26.2 | 18.4 - 34.0 | 25.7 | NS |
|  | 65+ | 8.6 | 1.8 | 0.0 - 3.8 | 10.8 | 6.8 - 14.8 | 490.9 | < 0.001 |
| Males | | 4.4 | 3.1 | 2.0 - 4.2 | 3.4 | 2.2 - 4.6 | 9.7 | NS |
|  | 18-24 | 0.4 | 0.6 | 0.0 - 2.2 | 0.6 | 0.0 - 2.2 | 0.2 | NS |
|  | 25-34 | 2.3 | 1.1 | 0.0 - 2.9 | 2.1 | 0.0 - 4.2 | 82.2 | NS |
|  | 35-44 | 4.7 | 3.4 | 0.5 - 6.2 | 2.9 | 0.4 - 5.4 | -13.7 | NS |
|  | 45-54 | 7.9 | 4.1 | 0.9 - 7.4 | 5.6 | 1.9 - 9.3 | 35.5 | NS |
|  | 55-64 | 7.3 | 5.7 | 1.7 - 9.7 | 5.1 | 1.5 - 8.7 | -10.2 | NS |
|  | 65+ | 3.4 | 3.5 | 0.6 - 6.3 | 3.5 | 0.9 - 6.1 | 1.4 | NS |
| Public hospital | | 8.5 | 7.2 | 6.0 - 8.4 | 5.9 | 4.9 - 6.9 | -17.9 | NS |
|  | 18-24 | 3.1 | 3.4 | 1.0 - 5.9 | 1.6 | 0.0 - 3.4 | -53.4 | NS |
|  | 25-34 | 8.0 | 8.0 | 5.1 - 10.9 | 4.3 | 2.2 - 6.4 | -46.4 | NS |
|  | 35-44 | 12.5 | 11.2 | 7.7 - 14.7 | 6.8 | 4.1 - 9.5 | -39.4 | NS |
|  | 45-54 | 13.7 | 9.7 | 6.3 - 13.0 | 11.3 | 7.8 - 14.9 | 17.1 | NS |
|  | 55-64 | 10.1 | 8.3 | 5.0 - 11.7 | 7.5 | 4.5 - 10.5 | -9.6 | NS |
|  | 65+ | 3.0 | 2.1 | 0.5 - 3.6 | 3.3 | 1.6 - 5.0 | 60.7 | NS |
| Females | | 12.5 | 10.3 | 8.4 - 12.2 | 9.6 | 7.8 - 11.4 | -6.8 | NS |
|  | 18-24 | 4.8 | 4.4 | 0.4 - 8.3 | 3.3 | 0.0 - 6.8 | -24.2 | NS |
|  | 25-34 | 12.7 | 13.2 | 7.8 - 18.6 | 7.2 | 3.4 - 11.1 | -45.3 | NS |
|  | 35-44 | 18.7 | 17.5 | 11.3 - 23.7 | 11.7 | 6.7 - 16.6 | -33.1 | NS |
|  | 45-54 | 20.1 | 12.3 | 6.9 - 17.7 | 17.0 | 10.8 - 23.1 | 38.0 | NS |
|  | 55-64 | 14.1 | 11.6 | 6.0 - 17.2 | 12.7 | 7.2 - 18.2 | 9.6 | NS |
|  | 65+ | 3.9 | 2.3 | 0.1 - 4.5 | 5.1 | 2.3 - 8.0 | 120.5 | NS |
| Males | | 4.4 | 4.0 | 2.7 - 5.3 | 2.1 | 1.2 - 3.0 | -47.5 | NS |
|  | 18-24 | 1.5 | 2.5 | 0.0 - 5.5 | 0.0 | 0.0 - 0.8 | -100.0 | NS |
|  | 25-34 | 3.3 | 2.8 | 0.2 - 5.4 | 1.4 | 0.0 - 3.1 | -51.4 | NS |
|  | 35-44 | 6.2 | 4.8 | 1.4 - 8.1 | 1.8 | 0.0 - 3.8 | -61.8 | NS |
|  | 45-54 | 7.1 | 7.0 | 2.9 - 11.1 | 5.6 | 1.9 - 9.3 | -20.3 | NS |
|  | 55-64 | 6.0 | 5.0 | 1.2 - 8.8 | 2.1 | 0.0 - 4.6 | -57.2 | NS |
|  | 65+ | 2.0 | 1.7 | 0.0 - 3.8 | 1.2 | 0.0 - 2.8 | -29.5 | NS |
| Private hospital | | 132.2 | 82.7 | 78.8 - 86.6 | 159.0 | 153.7 - 164.3 | 92.3 | < 0.001 |
|  | 18-24 | 63.3 | 36.5 | 28.8 - 44.1 | 88.3 | 76.4 - 100.2 | 142.1 | < 0.001 |
|  | 25-34 | 149.1 | 82.2 | 72.9 - 91.4 | 211.3 | 197.2 - 225.4 | 157.1 | < 0.001 |
|  | 35-44 | 220.5 | 136.8 | 124.6 - 149.0 | 259.7 | 243.6 - 275.7 | 89.8 | < 0.001 |
|  | 45-54 | 207.3 | 124.8 | 112.9 - 136.6 | 239.6 | 223.5 - 255.7 | 92.0 | < 0.001 |
|  | 55-64 | 119.2 | 91.8 | 80.9 - 102.7 | 120.0 | 108.2 - 131.7 | 30.6 | < 0.001 |
|  | 65+ | 22.7 | 19.0 | 14.7 - 23.4 | 21.0 | 16.9 - 25.1 | 10.6 | NS |
| Females | | 204.5 | 122.2 | 115.5 - 128.9 | 252.2 | 242.8 - 261.6 | 106.4 | < 0.001 |
|  | 18-24 | 110.3 | 60.8 | 46.6 - 75.0 | 154.1 | 131.3 - 176.8 | 153.3 | < 0.001 |
|  | 25-34 | 243.5 | 129.0 | 112.6 - 145.3 | 352.0 | 326.4 - 377.7 | 173.0 | < 0.001 |
|  | 35-44 | 344.8 | 208.0 | 186.9 - 229.0 | 409.2 | 380.8 - 437.6 | 96.8 | < 0.001 |
|  | 45-54 | 317.7 | 185.6 | 165.3 - 205.8 | 377.8 | 349.4 - 406.2 | 103.6 | < 0.001 |
|  | 55-64 | 178.0 | 128.6 | 110.4 - 146.7 | 185.5 | 165.1 - 205.9 | 44.3 | < 0.001 |
|  | 65+ | 29.4 | 20.9 | 14.6 - 27.2 | 30.2 | 23.5 - 36.9 | 44.4 | NS |
| Males | | 57.2 | 41.8 | 37.8 - 45.8 | 62.7 | 57.9 - 67.5 | 50.0 | < 0.001 |
|  | 18-24 | 19.0 | 13.1 | 6.6 - 19.7 | 27.2 | 17.9 - 36.5 | 106.9 | NS |
|  | 25-34 | 54.6 | 35.4 | 26.7 - 44.1 | 70.7 | 59.0 - 82.4 | 99.6 | < 0.001 |
|  | 35-44 | 94.8 | 64.1 | 52.2 - 76.0 | 108.4 | 93.7 - 123.1 | 69.1 | < 0.001 |
|  | 45-54 | 93.5 | 62.5 | 50.5 - 74.5 | 98.0 | 83.2 - 112.8 | 56.8 | < 0.001 |
|  | 55-64 | 57.9 | 54.2 | 42.2 - 66.2 | 51.6 | 40.5 - 62.8 | -4.6 | NS |
|  | 65+ | 15.1 | 16.8 | 10.8 - 22.9 | 10.6 | 6.2 - 14.9 | -37.1 | NS |
| Metropolitan | | 142.7 | 101.0 | 95.3 - 106.7 | 151.6 | 145.0 - 158.2 | 50.1 | < 0.001 |
|  | 18-24 | 100.0 | 66.4 | 51.5 - 81.4 | 128.2 | 107.6 - 148.8 | 93.0 | < 0.001 |
|  | 25-34 | 144.5 | 93.2 | 81.4 - 105.0 | 181.3 | 165.5 - 197.0 | 94.5 | < 0.001 |
|  | 35-44 | 217.1 | 154.1 | 138.1 - 170.0 | 221.2 | 203.2 - 239.2 | 43.6 | < 0.001 |
|  | 45-54 | 209.7 | 136.2 | 120.3 - 152.2 | 213.8 | 194.5 - 233.1 | 57.0 | < 0.001 |
|  | 55-64 | 128.0 | 114.8 | 98.4 - 131.2 | 108.2 | 93.3 - 123.0 | -5.8 | NS |
|  | 65+ | 26.4 | 23.0 | 16.3 - 29.8 | 19.9 | 14.3 - 25.4 | -13.7 | NS |
| Females | | 216.1 | 146.9 | 137.2 - 156.6 | 235.3 | 223.6 - 247.0 | 60.2 | < 0.001 |
|  | 18-24 | 171.0 | 108.8 | 81.5 - 136.1 | 221.7 | 182.6 - 260.8 | 103.7 | < 0.001 |
|  | 25-34 | 231.2 | 144.1 | 123.3 - 164.9 | 295.3 | 266.8 - 323.9 | 104.9 | < 0.001 |
|  | 35-44 | 332.4 | 230.9 | 203.3 - 258.5 | 340.3 | 308.6 - 371.9 | 47.4 | < 0.001 |
|  | 45-54 | 314.0 | 198.7 | 171.5 - 225.9 | 327.1 | 293.5 - 360.6 | 64.6 | < 0.001 |
|  | 55-64 | 185.5 | 154.7 | 128.0 - 181.4 | 165.7 | 140.0 - 191.5 | 7.1 | NS |
|  | 65+ | 33.4 | 25.3 | 15.6 - 34.9 | 26.4 | 17.7 - 35.1 | 4.6 | NS |
| Males | | 66.9 | 53.5 | 47.5 - 59.5 | 65.6 | 59.3 - 71.9 | 22.6 | NS |
|  | 18-24 | 32.4 | 25.3 | 12.1 - 38.6 | 41.9 | 25.2 - 58.5 | 65.2 | NS |
|  | 25-34 | 57.9 | 42.4 | 31.0 - 53.8 | 67.4 | 53.6 - 81.2 | 58.9 | NS |
|  | 35-44 | 101.9 | 76.2 | 60.1 - 92.3 | 102.0 | 84.5 - 119.5 | 33.8 | NS |
|  | 45-54 | 102.9 | 72.7 | 56.0 - 89.4 | 98.2 | 79.5 - 116.9 | 35.1 | NS |
|  | 55-64 | 67.9 | 73.4 | 54.5 - 92.2 | 48.4 | 34.0 - 62.7 | -34.0 | NS |
|  | 65+ | 18.3 | 20.4 | 10.9 - 29.9 | 12.2 | 5.7 - 18.8 | -40.0 | NS |
| Regional | | 175.7 | 89.4 | 80.3 - 98.5 | 241.2 | 226.7 - 255.7 | 169.8 | < 0.001 |
|  | 18-24 | 74.4 | 33.9 | 14.2 - 53.6 | 116.6 | 80.7 - 152.6 | 244.1 | < 0.001 |
|  | 25-34 | 215.3 | 89.5 | 66.4 - 112.7 | 330.1 | 288.5 - 371.6 | 268.7 | < 0.001 |
|  | 35-44 | 311.6 | 145.0 | 116.6 - 173.3 | 428.3 | 380.3 - 476.3 | 195.5 | < 0.001 |
|  | 45-54 | 285.6 | 155.7 | 127.2 - 184.1 | 384.3 | 339.7 - 429.0 | 146.9 | < 0.001 |
|  | 55-64 | 158.8 | 93.3 | 70.4 - 116.1 | 183.8 | 153.5 - 214.1 | 97.1 | < 0.001 |
|  | 65+ | 28.8 | 20.3 | 11.3 - 29.3 | 36.5 | 25.8 - 47.2 | 79.7 | NS |
| Females | | 277.0 | 136.2 | 120.5 - 151.9 | 388.9 | 363.2 - 414.6 | 185.5 | < 0.001 |
|  | 18-24 | 130.0 | 52.0 | 16.6 - 87.4 | 213.4 | 143.3 - 283.4 | 310.2 | < 0.001 |
|  | 25-34 | 363.2 | 151.6 | 108.8 - 194.4 | 556.0 | 479.7 - 632.4 | 266.7 | < 0.001 |
|  | 35-44 | 501.7 | 233.8 | 183.1 - 284.5 | 696.3 | 610.1 - 782.5 | 197.9 | < 0.001 |
|  | 45-54 | 450.4 | 228.8 | 180.2 - 277.3 | 624.1 | 543.9 - 704.3 | 172.8 | < 0.001 |
|  | 55-64 | 241.8 | 139.6 | 100.2 - 179.0 | 281.7 | 229.2 - 334.1 | 101.8 | < 0.001 |
|  | 65+ | 37.9 | 25.8 | 12.0 - 39.5 | 56.7 | 38.3 - 75.0 | 120.0 | NS |
| Males | | 68.9 | 40.3 | 31.6 - 49.0 | 86.4 | 73.9 - 98.9 | 114.4 | < 0.001 |
|  | 18-24 | 21.8 | 16.9 | 0.0 - 37.4 | 26.3 | 1.4 - 51.2 | 55.7 | NS |
|  | 25-34 | 67.6 | 27.7 | 8.9 - 46.5 | 104.8 | 71.2 - 138.4 | 279.0 | < 0.001 |
|  | 35-44 | 115.8 | 53.6 | 28.4 - 78.8 | 152.2 | 110.8 - 193.6 | 184.1 | < 0.001 |
|  | 45-54 | 114.6 | 80.0 | 50.4 - 109.5 | 138.5 | 99.9 - 177.1 | 73.2 | NS |
|  | 55-64 | 71.4 | 45.6 | 22.2 - 68.9 | 80.4 | 51.2 - 109.6 | 76.6 | NS |
|  | 65+ | 18.3 | 14.0 | 2.7 - 25.2 | 13.6 | 3.7 - 23.5 | -2.6 | NS |
| Rural | | 94.8 | 61.2 | 52.9 - 69.5 | 116.2 | 104.9 - 127.5 | 89.9 | < 0.001 |
|  | 18-24 | 52.7 | 24.0 | 3.5 - 44.5 | 83.3 | 45.6 - 120.9 | 247.3 | < 0.001 |
|  | 25-34 | 118.3 | 65.5 | 41.8 - 89.2 | 162.0 | 127.9 - 196.1 | 147.4 | < 0.001 |
|  | 35-44 | 169.0 | 110.6 | 82.3 - 138.9 | 209.5 | 170.7 - 248.2 | 89.4 | < 0.001 |
|  | 45-54 | 147.5 | 94.3 | 70.0 - 118.7 | 172.3 | 138.7 - 205.9 | 82.7 | < 0.001 |
|  | 55-64 | 83.9 | 60.6 | 40.9 - 80.3 | 93.3 | 69.8 - 116.8 | 54.0 | NS |
|  | 65+ | 17.4 | 15.0 | 6.5 - 23.4 | 17.9 | 9.7 - 26.1 | 19.7 | NS |
| Females | | 149.9 | 91.3 | 76.9 - 105.7 | 193.7 | 173.1 - 214.3 | 112.2 | < 0.001 |
|  | 18-24 | 95.5 | 40.7 | 2.1 - 79.3 | 154.5 | 80.5 - 228.4 | 279.3 | < 0.001 |
|  | 25-34 | 199.3 | 103.5 | 61.5 - 145.6 | 295.1 | 230.0 - 360.1 | 185.0 | < 0.001 |
|  | 35-44 | 269.6 | 166.9 | 118.2 - 215.6 | 345.1 | 275.1 - 415.0 | 106.7 | < 0.001 |
|  | 45-54 | 230.5 | 146.1 | 103.4 - 188.7 | 277.8 | 217.8 - 337.8 | 90.2 | < 0.001 |
|  | 55-64 | 129.2 | 89.1 | 55.0 - 123.3 | 151.5 | 109.5 - 193.6 | 70.0 | NS |
|  | 65+ | 24.3 | 15.3 | 3.4 - 27.2 | 28.9 | 14.4 - 43.4 | 88.7 | NS |
| Males | | 37.2 | 30.0 | 21.5 - 38.5 | 35.8 | 26.6 - 45.0 | 19.3 | NS |
|  | 18-24 | 12.3 | 8.2 | 0.0 - 26.9 | 16.9 | 0.0 - 42.6 | 106.2 | NS |
|  | 25-34 | 36.3 | 27.0 | 4.6 - 49.3 | 28.2 | 7.2 - 49.3 | 4.8 | NS |
|  | 35-44 | 63.9 | 51.4 | 23.1 - 79.7 | 68.5 | 36.1 - 101.0 | 33.3 | NS |
|  | 45-54 | 60.7 | 40.8 | 17.4 - 64.3 | 63.4 | 33.6 - 93.1 | 55.2 | NS |
|  | 55-64 | 37.8 | 32.6 | 11.6 - 53.5 | 33.1 | 12.6 - 53.6 | 1.6 | NS |
|  | 65+ | 9.9 | 14.6 | 2.1 - 27.0 | 6.1 | 0.0 - 13.4 | -58.2 | NS |

| **Table S2.** **Procedure codes and classification by surgical intention** | | |
| --- | --- | --- |
| **Classification** | **Procedure code** | **Procedure description** |
| Primary procedures | |  |
|  | 30511-02 | Laparoscopic adjustable gastric banding [LAGB] |
|  | 30511-03 | Laparoscopic nonadjustable gastric banding [LNGB] |
|  | 30511-04 | Adjustable gastric banding [AGB] |
|  | 30511-05 | Nonadjustable gastric banding [NGB] |
|  | 30511-06 | Laparoscopic gastroplasty |
|  | 30511-07 | Endoscopic gastroplasty |
|  | 30511-08 | Gastroplasty |
|  | 30511-09 | Laparoscopic sleeve gastrectomy [LSG] |
|  | 30511-10 | Sleeve gastrectomy [SG] |
|  | 30511-13 | Laparoscopic gastric banding |
|  | 30511-14 | Gastric banding |
|  | 30512-00 | Gastric bypass |
|  | 30512-01 | Laparoscopic biliopancreatic diversion [LBPD] |
|  | 30512-02 | Biliopancreatic diversion [BPD] |
|  | 30512-03 | Laparoscopic gastric bypass |
|  | 90940-00 | Duodenal-jejunal bypass [DJ Bypass] |
|  | 90941-00 | Ileal interposition |
|  | 90950-00 | Insertion of gastric balloon |
|  | 90950-02 | Endoscopic insertion of device into stomach |
| Revision procedures | |  |
|  | 14215-01 | Adjustment of gastric band reservoir |
|  | 30511-11 | Laparoscopic revision of gastric band |
|  | 30511-12 | Revision of gastric band |
|  | 30514-01 | Revision of procedure for obesity |
|  | 31441-00 | Replacement of gastric band reservoir |
|  | 31441-01 | Removal of gastric band reservoir |
|  | 31590-00 | Revision of gastric band reservoir |
|  | 90942-00 | Removal of gastric band |
|  | 90942-01 | Laparoscopic removal of gastric band |
|  | 90942-02 | Endoscopic removal of gastric band |
|  | 90943-00 | Other procedures for obesity |
|  | 90943-01 | Other laparoscopic procedures for obesity |
|  | 90943-02 | Other endoscopic procedures for obesity |
|  | 90950-01 | Removal of gastric balloon |
|  | 90950-03 | Endoscopic removal of device from stomach |
|  | 90950-04 | Endoscopic revision of device in stomach |
| Endoscopic procedures | |  |
|  | 30511-07 | Endoscopic gastroplasty |
|  | 90942-02 | Endoscopic removal of gastric band |
|  | 90943-02 | Other endoscopic procedures for obesity |
|  | 90950-00 | Insertion of gastric balloon |
|  | 90950-01 | Removal of gastric balloon |
|  | 90950-02 | Endoscopic insertion of device into stomach |
|  | 90950-03 | Endoscopic removal of device from stomach |
|  | 90950-04 | Endoscopic adjustment of gastric balloon |

| **Table S3.** **Local health districts and classifications by geographical area** | |
| --- | --- |
| **Geographical area** | **Local Health District (LHD)** |
| Metropolitan |  |
|  | Nepean Blue Mountains |
|  | Northern Sydney |
|  | South Eastern Sydney |
|  | South Western Sydney |
|  | Sydney |
|  | Western Sydney |
| Regional |  |
|  | Central Coast |
|  | Hunter New England |
|  | Illawarra Shoalhaven |
| Rural |  |
|  | Far West |
|  | Mid North Coast |
|  | Murrumbidgee |
|  | Northern NSW |
|  | Southern NSW |
|  | Western NSW |
